# RESEARCH ON SUPPORT FOR ELDER CARE IN DIFFERENT CULTURES AND SETTINGS

**DOI:** 10.1093/geroni/igad104.1595

**Published:** 2023-12-21

**Authors:** Lin Jiang, Ning Zhang

## Abstract

This cross-culture symposium consists of four studies that examined the interventions, patterns, technology, and challenges for elder care across settings in the U.S., China, and other countries. The first two papers focus on caregivers for patients with Alzheimer’s disease and related dementia (ADRD). The paper from the U.S. fills that gap by examining the information and communications technology utilization in relation to mental health among Mexican American informal caregivers for persons with ADRD. The findings suggest a need to explore further different types of ICT and their effects on Mexican American informal caregivers’ mental health. To help t reduce social isolation and loneliness, we may consider choosing an appropriate ICT based on their situation and culture. The paper from China highlights ADRD informal caregivers’ needs and struggles, which were ignored by the Chinese public. Also, the paper examined a model for healthcare professionals at inpatient and outpatient clinics to provide support to caregivers medically and emotionally. The third paper is a systematic review summarizing the latest evidence regarding the importance of using technology among people with dementia and their caregivers as an intervention. The fourth paper broadens our horizon by using a multinational comparison perspective to analyze research on pattern recognition and innovation of home-based care under the change of family structure in China. After individual presentations are done, one discussant will summarize shared themes in these papers and provide critical feedback.

